# No Jab, No Job? Ethical Issues in Mandatory COVID-19 Vaccination of Healthcare Personnel

**DOI:** 10.1136/bmjgh-2020-004877

**Published:** 2021-02-17

**Authors:** Rachel Gur-Arie, Euzebiusz Jamrozik, Patricia Kingori

**Affiliations:** 1 Berman Institute of Bioethics, Johns Hopkins University, Baltimore, Maryland, USA; 2 Oxford-Johns Hopkins Global Infectious Disease Ethics Collaborative, Oxford, UK, Baltimore, Maryland, USA; 3 Wellcome Centre for Ethics and Humanities, University of Oxford, Oxford, UK

**Keywords:** COVID-19, vaccines, public health, health policy, environmental health

**Summary box**

- Mandating COVID-19 vaccination of healthcare personnel (HCP) could maximise vaccine uptake, but risks exacerbating breakdowns in trust between HCP and their institutions.
- Ethical arguments for mandating COVID-19 vaccination of HCP appeal to their duties to ‘do no harm’ and to care for patients, but the fulfilment of these duties requires a safe working environment.
- We argue for policies aimed at strengthening HCP’s trust in healthcare systems by addressing HCP concerns, including the institutional factors that have put them at risk of infection throughout the COVID-19 pandemic, before considering a COVID-19 vaccine mandate.

## Introduction

The COVID-19 pandemic continues, but its potential end is in sight. The first COVID-19 vaccines have been distributed.^1 2^ Multiple vaccine allocation plans rooted in fair, just and equitable global vaccine allocation aim to maximise the benefits of vaccination programmes.^3–6^ One common factor in COVID-19 vaccine allocation plans to date is the prioritisation of healthcare personnel (HCP). We define HCP as all frontline workers at healthcare settings including but not limited to: physicians, nurses, physicians’ assistants, laboratory technicians, students on rotation, administrative staff, cleaning personnel and security staff. Though hierarchies of vaccine prioritisation among different groups of HCP have been proposed, this analysis focuses on HCP generally as a population.^4^ Prioritising HCP to receive COVID-19 vaccines is relatively uncontroversial and commonly justified by HCP’s right to be protected from occupational infection, the need to maintain healthcare staffing, and/or the protection of patients from being infected by HCP.^7^

The pandemic emergency has exacerbated shortages in healthcare staffing and resources,^8^ heightening the importance of protecting HCP. Vaccinating HCP against COVID-19 first could therefore help to maximise the benefit of a limited resource,^9^ assuming that vaccinated HCP continue to care for patients, including those infected with COVID-19. Vaccinated HCP may also impose less risk of disease transmission to patients, as documented in other infectious disease contexts including influenza and hepatitis B.^10 11^ All of these justifications are arguably related to HCP’s professional commitment to protect patients.

## How complicated can vaccinating HCP against COVID-19 be?

On face value, vaccinating HCP against COVID-19 first sounds simple: healthcare systems will receive COVID-19 vaccines, distribute them to their HCP, and HCP will be vaccinated. Though such a scenario sounds plausible, vaccine hesitancy among the general public and HCP was rising globally at record rates even before COVID-19.^12 13^ Previous research has documented the prevalence of vaccine hesitancy among HCP regarding COVID-19 vaccines.^14–17^ Potential contributors to COVID-19 vaccine hesitancy among HCP include safety concerns, doubts about effectiveness (in terms of individual protection and/or reduction of transmission), and perceived low risks of infection among HCP who do not treat patients with COVID-19.^14^ Similar factors contribute to vaccine hesitancy towards seasonal influenza vaccination among HCP,^18^ which has been a documented challenge in raising seasonal influenza vaccine uptake among HCP in some settings for decades, despite established evidence of safety and effectiveness.^18 19^ This raises the question: what kinds of policies aimed at increasing COVID-19 vaccine uptake among HCP would be ethically justifiable? Overarching consensus to prioritise HCP to receive COVID-19 vaccines is undoubtedly an achievement in itself, given the many other subpopulations with competing claims for priority. If vaccines primarily reduce severe disease (rather than transmission), there might arguably be a stronger ethical case for focusing vaccine distribution efforts to older individuals with comorbidities, for example. Even if it is agreed that HCP should be prioritised, there has been little discussion of HCP vaccine implementation policy, especially if voluntary vaccine uptake by HCP proves to be lower than expected. In this paper, we ask: how should HCP prioritisation be implemented?

## Exploring COVID-19 Vaccine Policy Options

The ethical acceptability of vaccination policies depends on factors including disease severity, vaccine effectiveness, safety and target population(s), as well as social, cultural and political considerations.^20^ Policy should attempt to draw on current evidence, attempt to manage residual uncertainties, and prepare for future developments.^21^ On one end of the vaccine policy spectrum are less restrictive options—opt-in, voluntary recommendations, and so on—while on the other end, there are more restrictive options—compulsory mandates backed with legal and financial penalties, with non-compliance potentially resulting in imprisonment.^22^ The current emergency arguably leaves little room for low vaccine uptake if we wish to reap the maximum benefits of vaccination, assuming adequate safety and effectiveness. Yet, vaccine hesitancy and logistical challenges pose significant threats to vaccine uptake generally and among HCP.

Vaccine mandates, though the most intrusive form of vaccine policy, have been shown to yield high vaccine uptake among HCP.^23^ For this reason, there has been heightened interest in mandating COVID-19 vaccines, once available.^24 25^ Public health ethics principles suggest that mandatory vaccination policies in adults should usually be the ‘last resort’.^26^ Mandatory policies have also been implemented for other public health measures when clear evidence suggests significant harm to others, such as prohibiting smoking in public areas and driving while under the influence of alcohol.^26^ Historically, mandatory vaccination policies have been more widely accepted in emergency situations due to outbreaks that posed an imminent threat due to consistent low vaccine uptake resulting from voluntary policies, especially in healthcare and educational settings.^22 27^

Mandatory influenza vaccination policies of HCP in healthcare sttings have gained popularity in some countries due to low vaccine uptake among HCP and heightened evidence supporting the influenza vaccine’s ability to reduce influenza infection and disease severity.^23^ Such policies consistently yield HCP influenza vaccine uptake rates above 90% while maintaining medical, philosophical, and religious exemptions.^23^ It is important to distinguish between government-implemented vaccine mandates and employer-mandated vaccine mandates. For example, the US Supreme Court case of *Jacobson* upheld mandatory smallpox vaccination at the government level for adults in the state of Massachusetts during a smallpox outbreak in the early 1900s.^24^ Institution-level mandates can be characterised by healthcare settings that mandate influenza vaccination of HCP when legally, politically and culturally permissible.^25^,^23^,^23^ The line between government-mandated and institution-mandated vaccine policies may be blurred when centralised national healthcare systems are in place and healthcare institutions are essentially extensions of government. As a result, ethical arguments for or against COVID-19 vaccine mandates should be sensitive to the policy level at which they are being implemented. On this note, while relevant precedents for mandating COVID-19 vaccination may exist, there might be more uncertainty regarding long-term effectiveness (including with regard to prevention of transmission) and safety of COVID-19 vaccines than the seasonal influenza vaccine, for example.^28^ Given the consensus prioritisation of HCP to get vaccinated against COVID-19, should vaccination of HCP be mandatory?

## The Case for and Against Mandating COVID-19 Vaccination of HCP

Mandatory vaccination of HCP might arguably involve justifiable limitations on HCP autonomy to ensure the fulfilment of certain professional responsibilities. On the one hand, HCP regularly work with and treat vulnerable populations, including older and immunocompromised individuals, heightening the importance of infection prevention including via uptake of safe and effective vaccines where these prevent transmission to patients.^29^ On the other hand, some HCP hold negative attitudes toward vaccines that contribute to low vaccine uptake.^30^ These negative attitudes seem to remain among HCP regarding COVID-19 vaccines.^15^ Research suggests additional concerns among HCP specifically regarding: COVID-19 vaccines (including insufficient safety and efficacy data and long-term side effects) and breakdowns of trust between HCP and institutions (due to inadequate personal protective equipment [PPE] and concerns that getting vaccinated against COVID-19 will be linked to requirements to work with COVID-19 patients).^15–17^

There has been a recent increase in seasonal influenza vaccination mandates for HCP based on their (1) obligation to ‘do no harm’, (2) professional duty to prioritise patients’ interests (‘duty to care’), and (3) perceived obligation to set a good example for the public.^29^ Some or all of this logic may be adopted as the justification for mandating COVID-19 vaccines to HCP once they become available. Nevertheless, there are important questions surrounding the basis of HCP’s commitment to ‘do no harm’ and the duty to care in the context of getting vaccinated against COVID-19.

## ‘Do no harm’

There is a prima facie case to mandate COVID-19 vaccination for HCP on the basis of HCP’s responsibility not to harm their patients.^31^ After all, protecting patients is one of the key justifications for requiring HCP to be vaccinated or show immunity against other documented occupational threats such as hepatitis B, measles, mumps, rubella, diphtheria and pertussis.^31^ Based on research conducted in healthcare settings, there is growing evidence that superspreading events may be a typical feature of COVID-19 transmission, and that HCP may be involved in such events.^32^ Superspreading COVID-19 events in healthcare settings have been documented in healthcare settings globally.^32 33^ Early in the pandemic, HCP accounted for 17% of COVID-19 cases in Argentina, suggesting the potential of healthcare settings to be nodes for infection and HCP as a superspreading population, consistent with data from the Lombardy region in Italy.^33^ Reducing superspreading events among HCP through maximising COVID-19 vaccine uptake via a mandate for a vaccine that prevents transmission could in turn help to reduce superspreading events in healthcare settings, benefiting vulnerable and/or immunocompromised populations that many HCP are focused on treating. Nevertheless, appeals to HCP’s commitment to do no harm when mandating COVID-19 vaccines are weakened by considering the systemic failures of many healthcare systems to protect HCP and patients from healthcare-associated infections in the first place.

Public health should arguably strive to implement the least restrictive intervention when possible,^26^ yet vaccine mandates are the most restrictive, intrusive form of vaccine policy. Ethical debate on vaccine mandates consistently suggests that unless all other reasonable means have failed (or are likely to fail) to increase vaccine uptake and/or reduce disease transmission by other means to an acceptable level, mandates should not be implemented.^21 22 34^ This requires institutions to consider whether they have pursued all possible interventions and support mechanisms for preventing infection even without a vaccine intervention. The pandemic has been characterised by shortages of PPE for HCP and overcrowded hospitals.^35 36^ As the pandemic continues, there is increasing evidence suggesting the effectiveness of PPE in preventing COVID-19 in healthcare settings.^37 38^ HCP commit to do no harm to their patients (including by taking steps to avoid infecting them), but their ability to fulfil this duty is frequently impaired by a lack of PPE and other occupational health protections including adequate uncrowded space for staff to take breaks and appropriate hospital ventilation.^39^ Moreover, if all such reasonable systemic protections were adequate, it is not clear that the risk of spread from HCP to patients would be unacceptably high—with or without vaccination. Under this analysis, mandating COVID-19 vaccination for HCP would not be ethically permissible insofar as the less coercive measure of providing proper PPE and other protections to HCP has not been fulfilled.

Critical analysis of the institutional responsibility to ‘do no harm’ has been overshadowed by the hero narrative associated with HCP’s bravery and dedication to work throughout the COVID-19 pandemic.^40^ However, the appropriateness of the ‘hero narrative’ weakens when institutions and healthcare systems fail to protect HCP’s health and well-being, especially when effective interventions are available but not implemented. This has arguably undermined trust between HCP and their institutions and perhaps also trust in the safety of COVID-19 vaccines. Institutions and healthcare systems which consider adopting a COVID-19 vaccine mandate among HCP should not ignore this history and context, in which HCP’s occupational conditions have consistently put them at higher risk of contracting and spreading COVID-19. Consequently, the failed institutional responsibility to assist HCP in the fulfilment of their duties to ‘do no harm’ weakens appeals to such duties in the justification of vaccine mandates.

## Duty to care

HCP’s duty to care for, protect, and prevent infection in patients has received increased attention, particularly in the context of COVID-19, as well as in the context of HIV/AIDS, SARS, pandemic influenza, and Ebola.^41^ ^42^ The notion that HCP consent to caring for patients, even if this puts HCP at significant risks of infection and even death, is expressed through implicit social contracts (HCP receive special privileges in society, and are expected to provide healthcare when necessary in return) and in professional codes of conduct.^41 43^ Calls for COVID-19 vaccine mandates for HCP made on the basis of HCP’s duty to care draw on the utilitarian notion that requiring vaccination would allow maximum benefit to the public by keeping HCP healthy and working during the pandemic at the expense of HCP autonomy.^31^ However, the utilitarian argument in this context is arguably flawed, because it focuses primarily on the social value of HCP’s labour. There are non-coercive interventions that can effectively combat COVID-19, such as widespread voluntary vaccination and non-pharmaceutical interventions in the general population. Such alternative interventions may achieve greater overall benefits including, indirectly, a reduction of healthcare-acquired infections, without specifically mandating HCP to get vaccinated against COVID-19. In this case, unless supply of COVID-19 vaccines is highly limited and there is good reason to think that enforced HCP vaccination produces maximum overall benefit, an HCP mandate could be even less justifiable.

Concerns about this frame of thinking expand beyond questions surrounding HCP autonomy, vaccine safety, and effectiveness. HCP may find themselves working in *even more* dangerous environments due to their vaccination status. They might justifiably fear, for example, being sent to work in situations without appropriate protections because of their assumed immunity and privilege of being vaccinated. In other words, mandating COVID-19 vaccination to HCP could unintentionally (or even intentionally) require HCP to accept more risky choices with already-limited institutional support and resources,^44^ ultimately leaving them potentially *worse off* than they were when unvaccinated. HCP have overwhelmingly displayed their commitment to their duty of care in recent months. However, HCP’s duty to care is not infinite—its limits are revealing themselves as the pandemic progresses.^45 46^

## Conclusions

COVID-19 vaccination of HCP has begun. The COVID-19 pandemic has emphasised the importance and need for creativity when protecting HCP’s occupational health.^47^ COVID-19 vaccine mandates could be perceived to be a ‘magic bullet’ measure to implement in order to maximise vaccine uptake among HCP. However, even if remaining uncertainties regarding safety and effectiveness of the vaccine are addressed, healthcare institutions, including universities or academic healthcare settings where HCP work, should not be too hasty to mandate HCP compliance with COVID-19 vaccination without weighing ethical concerns.^48^ Healthcare institutions should instead proceed by gaining the trust of HCP and strengthening safety protections to prevent what could be a strong resistance by HCP in certain settings.^31^ Institutions should start developing vaccination policies early, beginning with robust educational campaigns to promote voluntary vaccination,^49^ while realising that expecting HCP’s unquestioning acceptance and trust in COVID-19 vaccines may currently be unrealistic. Policymakers should navigate how to make the best decision for HCP and patients alike from appropriate social, cultural and political perspectives. They should also consider how to respond to potential negative impacts on individual HCP and wider public perceptions resulting from a mandatory policy. Herein lies the tension between wanting to prepare HCP for a potential COVID-19 vaccine mandate in order to avoid backlash, while respecting HCP’s understandable scepticism given their lived experiences on the frontlines of this pandemic. Reconciling this tension requires a willingness to both engage with HCP’s lived experiences and take their ethical concerns seriously.

## Data Availability

There are no data in this work.
